# Potato Cyst Nematodes: A New Threat to Potato Production in East Africa

**DOI:** 10.3389/fpls.2020.00670

**Published:** 2020-05-25

**Authors:** Harrison Mburu, Laura Cortada, Solveig Haukeland, Wilson Ronno, Moses Nyongesa, Zachary Kinyua, Joel L. Bargul, Danny Coyne

**Affiliations:** ^1^International Centre of Insect Physiology and Ecology (icipe), Nairobi, Kenya; ^2^East Africa Hub, International Institute of Tropical Agriculture (IITA), Nairobi, Kenya; ^3^Department of Biochemistry, Jomo Kenyatta University of Agriculture and Technology, Juja, Kenya; ^4^Nematology Section, Department of Biology, Ghent University, Ghent, Belgium; ^5^Biotechnology and Plant Health Division, Norwegian Institute for Bioeconomy Research, Ås, Norway; ^6^Food and Agriculture Organization (FAO), Nairobi, Kenya; ^7^Kenya Agricultural and Livestock Research Organization (KALRO), Nairobi, Kenya

**Keywords:** EPPO, *Globodera rostochiensis*, *Globodera pallida*, Kenya, smallholder farmers, potato seed systems, cv. Shangi

## Abstract

Potato cyst nematodes (PCN), such as *Globodera rostochiensis* and *Globodera pallida*, are quarantine restricted pests of potato causing major yield and financial losses to farmers. *G. rostochiensis* was first reported from Kenya’s key potato growing area in 2015. We sought to determine the diversity, prevalence and distribution of PCN species across the country by conducting a country-wide survey between 2016 and 2018, which included a more focused, follow-up assessment in three key potato growing counties. A total of 1,348 soil samples were collected from 20 potato growing counties. Information regarding local potato farming practices, potato cultivar use, their diversity and availability was also recorded. PCN cysts were obtained from 968 samples (71.8%) in all the counties surveyed, with Nyandarua County recording the highest PCN field-incidence at 47.6%. The majority of PCN populations, 99.9%, were identified as *G. rostochiensis*, while *G. pallida* was recovered from just one field, in a mixed population with *G. rostochiensis.* Inconsistencies in PCR amplification efficiency was observed for *G. rostochiensis* using the recommended EPPO primers, compared with ITS primers AB28/TW81, indicating that this protocol cannot be entirely relied upon to effectively detect PCN. Egg density in Nyandarua County varied between 30.6 and 158.5 viable eggs/g soil, with an average egg viability of 78.9 ± 2.8% (min = 11.6%, max = 99.9%). The PCN-susceptible potato cultivar named Shangi was the most preferred and used by 65% of farmers due to its shorter dormancy and cooking time, while imported cultivars (Destiny, Jelly, Manitou, and Markies) with resistance to *G. rostochiensis* were used by 7.5% of farmers due to unavailability and/or limited access to seeds. Thus, most farmers preferred using their own farm-saved seeds as opposed to purchasing certified seeds. Establishing the distribution and prevalence of PCN and elucidating the local farming practices that could promote the spread of PCN is a necessary precursor to the implementation of any containment or management strategy in the country and ultimately across the region.

## Introduction

Potato is a valuable and nutritious staple crop, driving both food security and Growth Domestic Product (GDP) growth globally (Reader, 2009; Devaux et al., 2014). Approximately half of the world’s potato is produced in Asia, especially China, followed by Europe producing about a third (Scott and Suarez, 2012). Only maize is grown in more countries than potato. Africa produces about 7% of global potato output, mainly in Egypt and South Africa (Devaux et al., 2014). Potato production in the East and Central Africa highlands offers great promise despite substantial fluctuations in yields recorded over the last two decades (FAOSTAT, 2018). Potatoes are a popular and valuable crop for both food security and income generation, competing well with maize in the subtropical climates at higher altitudes. Under these conditions, year-round production can be possible, often with at least two seasons per annum. In recent years, however, yields have shown notable declining trends, mainly attributed to major disease outbreaks, inappropriate cropping practices by farmers, substandard seed quality and lack of organized market infrastructure for produce (Janssens et al., 2013; Kaguongo et al., 2014). Emerging markets for processed potatoes (e.g., chips, crisps, starch) furthermore, have increasingly focused attention on potatoes, with rising demand from the fast food industry and processing for added economic value (Abong and Kabira, 2013). Processed potatoes, however, also demand high levels of quality, which can be difficult to sustain in the face of high pest and disease pressures (Janssens et al., 2013; Kaguongo et al., 2014). Thus, any action to improve potato production will have a considerable impact on food security and income in these countries (Scott et al., 2013; Haverkort and Struik, 2015; FAO, 2017b; Okello et al., 2017; Harahagazwe et al., 2018).

In Kenya, potato is the second most important staple food crop after maize and valued at ∼$500 million USD annually (CIP, 2019). About 800,000 Kenyans directly benefit from potato production, while across the whole value chain about 2.5 million people receive income from potato (Abong and Kabira, 2013). However, in Kenya, yields have declined and currently average 9–10 t/ha, much below the potential of 20–40 t/ha (Kiptoo et al., 2016; VIB, 2019), and as reflected across the region attributed to factors listed above. The situation is not helped by the emergence of new pests and diseases, such as the recently detected potato cyst nematodes (PCNs) *Globodera rostochiensis* and *G. pallida* (Mwangi et al., 2015; Mburu et al., 2018). PCNs are subject to strict quarantine regulations in over 100 countries (EPPO, 2017) and are globally considered as the most important pests threatening potato production but are all too often overlooked in less developed countries (Coyne et al., 2018; Niere and Karuri, 2018). The quarantine status of PCNs is, in part, related to their ability to produce quiescent structures known as cysts that consist of the hardened body of the females measuring ∼0.5 mm in diameter enclosing ∼300–500 eggs each. Cysts persist in the soil for long periods of up to 20 years, even in the absence of an appropriate host and can withstand extreme cold temperatures (−15°C) and prolonged desiccation periods (Folkertsma et al., 1997). This diapause cyst stage is broken after the appearance in the rhizosphere of specific root diffusates from a narrow range of hosts, mainly potato, although other solanaceous crops, such as tomato, eggplant or pepper can also stimulate egg hatch (Perry, 2002). The infective juveniles (J2) readily hatch in the presence of a host plant, yet in the absence of a suitable natural host cyst decline has been reported to be ∼0–20% in temperate regions, year-on-year (Devine et al., 1999). PCN decline under subtropical or tropical conditions in Africa is currently unknown. Yield losses associated with PCN will vary according to conditions, and earlier estimates in the EU suggest that for every 20 viable eggs/g soil ∼2.75 t/ha of potatoes are lost (Brown and Sykes, 1983). PCN is mainly spread through cyst-contaminated soil that adheres to the farm machinery, equipment or seeds.

The occurrence of PCN presents a key threat to potato production in Kenya, as well as to the entire East Africa region where potato features prominently as a food security crop or for income generation for millions of smallholder farmers (VIB, 2019). In order to mitigate the PCN threat in potato production, the establishment of the level of infestation, their geographical distribution, and the agronomic and social factors that could be influencing their distribution and spread are essential toward establishing and implementing a national management strategy. This may include exploring alternative sampling strategies, which would contribute to a detailed understanding of the spread of PCNs (Goeminne et al., 2011, 2015; Mimee et al., 2019). Consequently, this study presents the results of a countrywide survey, undertaken to determine the distribution of PCN and the potential damage it is causing in the major potato growing regions of Kenya. We additionally examined farmer potato production practices and how these will need to be taken into consideration for the implementation of future pest management strategies. The information we provide here should further help policy makers and regional stakeholders to make informed decisions related to PCN containment and mitigation.

## Materials and Methods

### Study Sites and Sampling Strategy

In an initial national survey, potato fields in 20 potato growing counties in Kenya were surveyed between 2016 and 2017, selecting approximately 1% of the potato production area per county, in line with the EU directive 2007/33/EC (Official Journal of European Union, 2007). A follow-up in-depth sampling was undertaken in 2018 in the major potato producing counties of Elgeyo Marakwet and Nyandarua as well as Taita Taveta, which presents significant potential for potato production (Jaetzold and Schmidt, 1983). A focused, in-depth study was also undertaken in Nyandarua County in order to estimate yield losses being experienced from PCN. Mean annual temperatures in surveyed counties ranged between 12.9 and 35°C, although lower temperatures of 2°C was recorded in some areas in Nyandarua County where occasional frosting can be experienced. For each sampled field the GPS coordinates were recorded, and a semi-structured questionnaire administered to capture farmer potato production practices (Supplementary Material). A composite soil sample of ∼1.5 kg comprising of 50 sub-samples (cores) was randomly collected from each field using a hand trowel from the top 30 cm of soil following a “zigzag” pattern (Coyne et al., 2018), and placed in a plastic container. When targeting certified potato seed farms (22 sites), ∼2.0 kg of composite soil samples were collected using the same sampling pattern as described above. Each sample was well secured, labeled, placed in a cooler-box and transported to *icipe* laboratories in Nairobi, Kenya, for processing.

### Isolation and Identification of Potato Cyst Nematodes

The soil samples were transported to the Kenya Plant Health Inspectorate Services (KEPHIS), where they were air-dried, thoroughly mixed, sieved to remove stones and debris before extraction of cysts from a 200 g sub-sample using the Fenwick can floatation method (EPPO, 2013b). Briefly, using a moderate but constant flow of water, each sample was washed through a 1 mm aperture sieve into the Fenwick can. Organic matter that passed through the 840 μm sieve in the can was collected from the overflow onto a 250 μm sieve. The sieve was backwashed and the final filtrate containing the cysts collected into 200 ml plastic beakers. Extractions were then collected on milk filter papers and air-dried. The cysts were individually handpicked using entomological forceps and counted using a Leica MZ12.5 dissection microscope. Cysts were placed in a 1 ml Eppendorf tube and stored at 4°C; the remaining soil was stored in case of further use.

Cysts recovered from samples were morphologically identified, based on the EPPO (2017) taxonomic guide, under a Leica MZ12.5 dissection microscope. The number of cysts (empty or containing eggs) positively identified as PCN were counted and recorded. About 1–10 cysts recovered from samples were subjected to molecular identification using modified EPPO multiplex-PCR protocols (EPPO, 2017; Mburu et al., 2018) and ITS primers (Joyce et al., 1994) for species identification. DNA amplification was carried out using ProFlex PCR systems^TM^ Base thermocycler (Applied Biosystems Life Technologies) with multiplex primers (PITSr3: 5’-AGC GCA GAC ATG CCG CAA-3’, PITSp4: 5’-ACA ACA GCA ATC GTC GAG-3’and ITS5 5’-GGA AGT AAA AGT CGT AAC AAG G-3’), which are species-specific for *G. pallida* (265 bp amplicon) and/or *G. rostochiensis* (434 bp amplicon) targeting the 18S rRNA gene and the internal transcribed spacer ITS1 region and ITS primers (TW81 5’-GTT TCC GTA GGT GAA CCT GC-3’ and AB28 5’-ATA TGC TTA AGT TCA GCG GGT-3’) targeting the ITS1-5.8S-ITS2 regions (1,100 bp amplicon). Each PCR reaction mixture (One Taq^®^ 2X Master Mix with standard buffer, New England Biolabs^®^ Inc., United States) contained 12.5 μl PCR master mix (One Taq^®^ 2X Master Mix with standard buffer), 1 μl of each primer (forward and reverse), 8.5 μl nuclease-free water and 2 μl of DNA (template) totaling 25 μl volume per reaction. PCR amplicons were electrophoresed through 2% agarose gel at 100V for 1 h to confirm successful amplification and size of the amplicons against a 100 bp ladder (New England Biolabs^®^, Inc., United States). For sequencing, species-specific PCR reactions were conducted using a singleplex approach with ITS5/PITSp4 primers for *G. pallida* and ITS5/PITSr3 for *G. rostochiensis*; PCR-amplicons were purified using the QIAquick PCR Purification Kit (Qiagen, United States) and sequenced by Sanger sequencing. DNA sequences were manually edited using BioEdit Sequence Alignment Editor (Hall, 1999). The edited sequences were analyzed using NCBI-BLAST tool and compared with previously isolated Kenyan isolates (Mwangi et al., 2015) and other *Globodera* spp. to generate a phylogenetic tree using Seaview software version 4.5.0 (Gouy et al., 2010).

### Incidence, Infestation and Yield Loss Assessment

A focused, more in-depth study was undertaken in Nyandarua County, which has the largest area of cultivated potato in Kenya. Data related to incidence and infestation level of PCN across surveyed farms were collected from five sub-counties (Kinangop, Kipipiri, Ol’Kalou, Ol’Joro Orok and Ndaragua). For each sub-county, the average PCN-infestation level was calculated as the mean number of cysts per 200 g soil recovered from all the farms visited. Incidence (%) at the county level was further determined as the number of fields where cysts were isolated from the total number of sampled farms and expressed as a percentage. Based on these results, counties were classified as low PCN-incidence (50% ≤ *X* < 70%), mid PCN-incidence (70% ≤ *X* < 90%) and high PCN-incidence (*X* ≥ 90%). Further classification for the in-depth study in Nyandarua County was undertaken at the sub-county level, where infestation levels were classified according to: 1 < *X* < 25 cysts, 26 < *X* < 110 cysts, 111 < *X* < 230 cysts, 231 < *X* < 495 cysts, and 496 < *X* < 985 cysts per 200 g of soil.

The impact of PCN on potato production was estimated using the Brown and Sykes (1983) regression line, which estimates that for every 20 viable eggs/g soil 2.75 t/ha of potato are lost to PCN. Therefore, PCN egg viability (EV) was assessed using a modified protocol, adapted from Faggian et al. (2012), as described below. Cyst infestation levels varied across the fields within the county, as was the number of fields with different levels of infestation. In order to assess a proportionate number of cysts from fields with different infestation levels, fields were grouped according to infestation level and then cysts were collected from the fields within each cluster group (minimum 10 and a maximum 50 cysts per field). The fields which had over 20 cysts per 200 g soil were grouped into four clusters and a total of 46 fields were included in the assessment: cluster 1 [fields with 200 g soil (*n* = 14)], cluster 2 (between 100 and 200 cysts (*n* = 11)], cluster 3 (between 50 and 100 cysts (*n* = 5)], and cluster 4 (between 20 and 50 cysts (*n* = 16)]. The samples were assessed for egg viability using Nile Blue stain, which stains dead eggs and/or juveniles (J2) (Ogiga and Estey, 1974), allowing the visual differentiation between dead (non-viable) and live (viable) J2s and eggs (non-stained) under the microscope. For the viability tests, cysts were handpicked and placed inside a modified 1.5 ml Eppendorf tube; the end of the tube was cut-off and a nylon mesh glued across the bottom. The tubes were placed inside a 24-well flat-bottomed culture plate (Falcon^®^, Thomas Scientific) with 1 ml of 0.01% Nile Blue stain and incubated in the dark at room temperature for 48 h. After incubation, the stain was carefully rinsed and replaced with 1 ml sterile distilled water. Cysts were then individually removed, placed on a glass slide and gently cut open to expose the contents (Faggian et al., 2012). The total number of J2s, viable and non-viable eggs were counted to determine cyst fertility (CF). Subsequently, cyst viability (CV%) was calculated as the total number of live J2s and viable eggs divided by the CF and expressed as a percentage (CV%).

Finally, in each field, soil infectivity (SI) was calculated as the mean number of viable eggs per cyst, multiplied by the total number of cysts extracted in 200 g soil and expressed as viable eggs/g soil. For each sub-county, the mean SI was calculated from all the studied fields. In each sub-county, potato yield losses (t/ha) directly attributable to PCN were estimated based on the SI damage threshold determined by Brown and Sykes (1983).

### Detection of Potato Cyst Nematode Resistance *H1* Gene

Molecular analyses was conducted on the most popular potato cultivar grown in Kenya, cv. Shangi (Sinelle, 2018) and four cultivars recently introduced from the EU (cv.s Destiny, Manitou, Markies, and Jelly) (NPCK, 2019) to determine the presence of the *H1* gene, which confers resistance to *G. rostochiensis*. The cv. Jelly, which is resistant to *G. rostochiensis* with *H1* gene (NPCK, 2017), was used as a positive control and cv. Desiree as the susceptible control. DNA was extracted from three leaves of 1-month old potato plants grown in the greenhouse at *icipe*, Nairobi. PCR amplification of the genomic region containing *H1* gene was conducted using 57R, TG689 and BCH markers (Park et al., 2018).

### Determining Farmer Practices and Preferences in Potato Production

To understand how current smallholder farming practices could be influencing the incidence and distribution of PCN, farmer interviews were conducted at the household level (HH) during field sampling. A semi-structured questionnaire was used to gather agronomic and socioeconomic data to complement the phytosanitary data obtained from the epidemiological survey. Data on farm size (acres), potato cultivars grown, potato seed source, and crop management strategies, e.g., rotation periods, use of alternate crops, etc. were collected. Enumerators were trained prior to conducting the interviews. Data were collected directly onto tablets and collated using Epicollect^® 1^ software.

### Data Analysis

The data was analyzed using Microsoft^®^ Excel^®^ Version 1911, mean and standard error of the means were calculated using the “psych” package, while the model of significance was detected by analysis of variance with a chi-square test and the pairwise comparison of the treatments undertaken using Tukey’s multiple comparison test using the “lsmeans” on R version 3.6.1.

## Results

### Incidence, Distribution and Identification of Potato Cyst Nematodes

**FIGURE 1:**
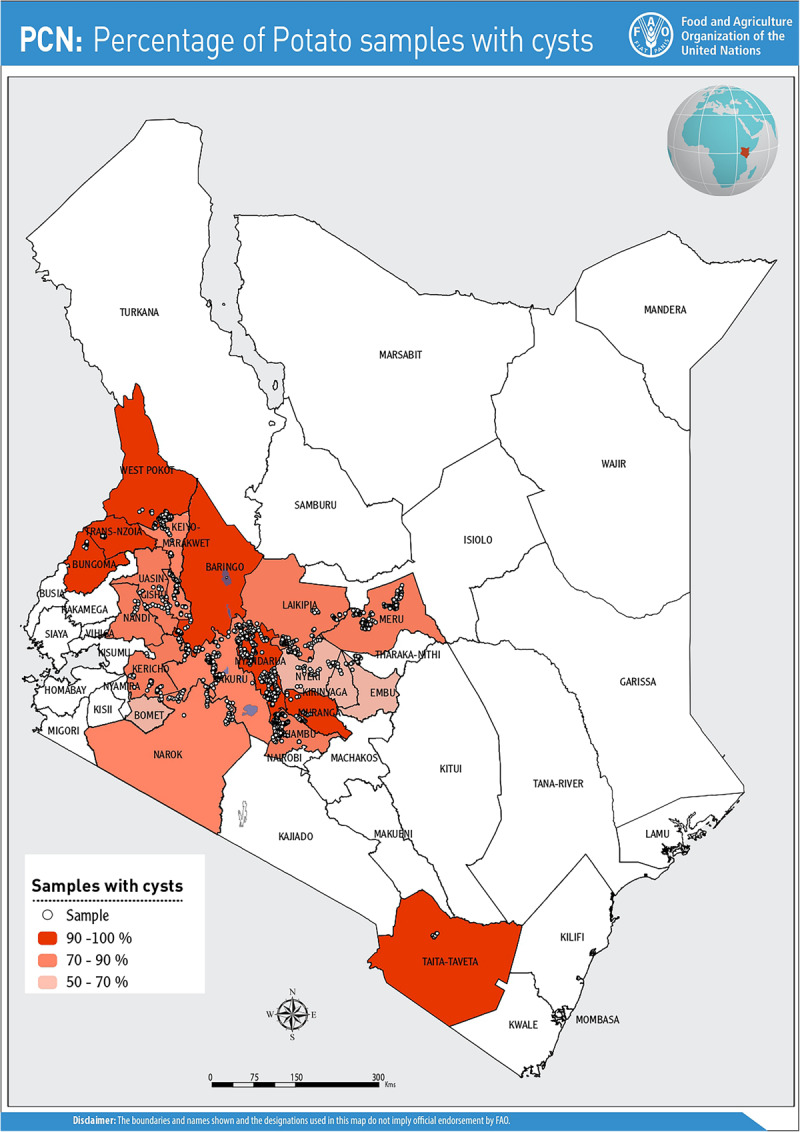
PCN distribution and incidence level classification across the 20 counties in Kenya.

Cysts were detected from farms in all the counties prospected and were recovered from a total of 902 field samples (82.8%) during the initial survey (2016–2017) and in 66 fields (25.6%) in the follow-up survey in 2018. Nyeri County presented the lowest incidence (53%), while counties such as Trans Nzoia, Taita Taveta and West Pokot had 100% incidence (Figure 1). Of the 22 certified seed farms sampled, 82% (*n* = 18/22) farms were infested with PCN.

### Detection and Molecular Identification of Potato Cyst Nematodes

**FIGURE 2:**
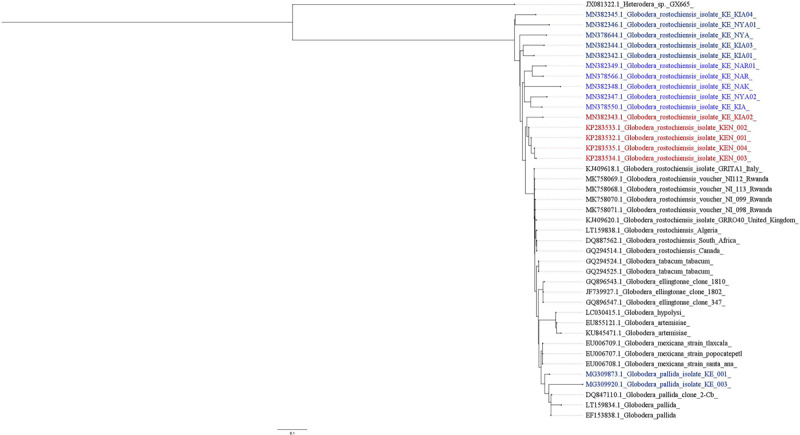
Phylogenetic relation of Kenyan PCN populations compared to those reported in Africa and rest of the world.

Of 968 samples that had sufficient numbers of cysts to enable molecular diagnosis, 170 (17.6%) were identified as *G. rostochiensis* (GenBank accession: MN378644.1, MN378550.1, MN378566.1, 382342.1 – MN382349.1) from a combination of both protocols. A relatively lower PCR amplification efficiency was observed with EPPO primers in PCN-species identification. However, cysts from 61 fields were amplified using ITS primers AB28/TW81 (Joyce et al., 1994), which produced amplicons in samples that previously did not yield any PCR products using the same DNA template with the EPPO primers. Samples amplifying with EPPO primers (ITS5/PITSr3) were recovered from 6 counties, 144 in Nyandarua (46.6%), 13 in Narok (20.3%), 6 in Kiambu (7.0%), 3 in Nakuru (2.2%), 3 in Taita Taveta (27.3%) and 1 in Nyeri (2.2%). PCR products obtained using ITS5/PITSr3 primers were sequenced (MN382342 to MN382345 Kiambu County, MN382346 and MN382347 Nyandarua County, and MN382348 and MN382349 Narok County) and analyzed using the NCBI-BLAST tool. Our findings show high sequence identity (%) ranging from 90 to 95% to *G. rostochiensis* populations from EU, Asia, and Northern and Southern Africa, including those previously described in Kenya (Figure 2).

### Detection of the *H1* Resistance Gene

The molecular analyses of potato cultivars confirmed the presence of the *H1* gene in cv.s Manitou, Markies, Destiny and Jelly (positive control) but not for cv. Shangi, indicating its susceptibility to *G. rostochiensis* (Table 1).

**TABLE 1:** Detection of *Globodera rostochiensis* (Ro1) *H1 resistance* gene in Kenyan grown potato cultivars.

**Cultivars**	**BCH primers**	**TG689 primers**	**57R primers**	**Classification of PCN resistance^1^**
Shangi	+	−	−	Susceptible (This article)
Manitou	+	+	+	Resistant
Markies	+	+	+	Resistant
Destiny	+	+	+	Resistant
Desireé	+	−	−	Susceptible control
Jelly	+	+	+	Resistant control

*^1^As referenced in the National Potato Council of Kenya NPCK (2019).*

### Potato Cyst Nematode Infestation and Yield Loss Assessment

**FIGURE 3:**
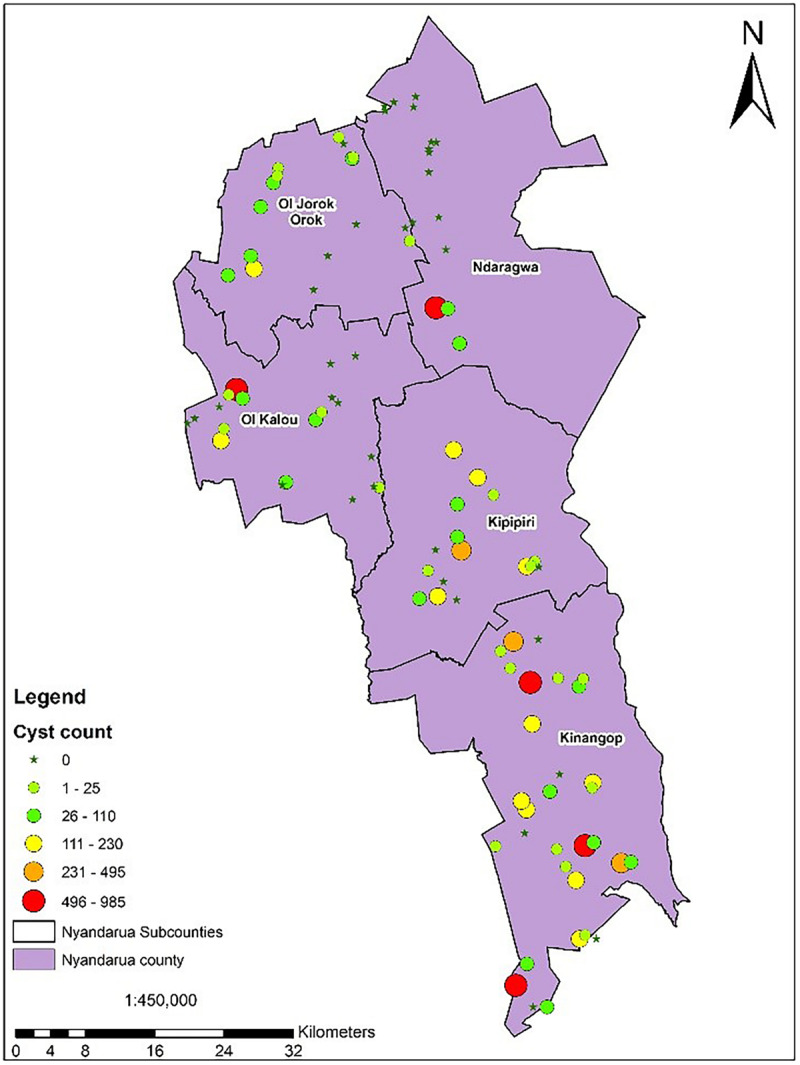
PCN infestation levels across the 5 sub-counties of Nyandarua indicating the “hot-spots” in red.

**FIGURE 4:**
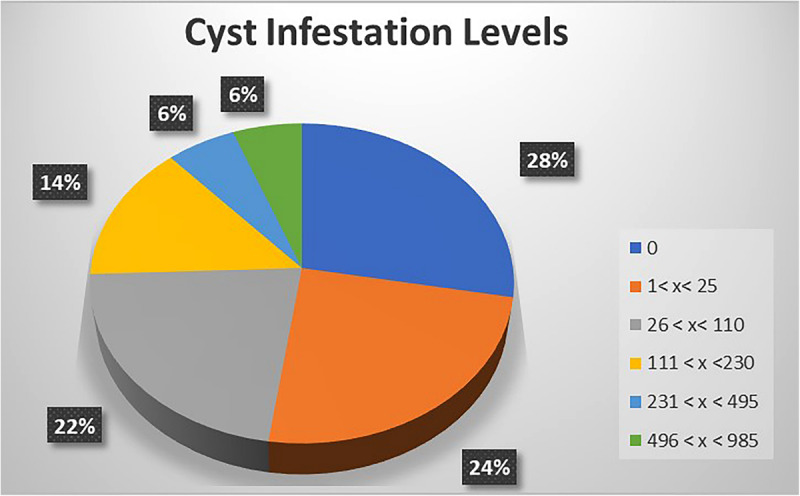
A representation of PCN cyst infestation levels across the 5 Sub-counties of Nyandarua County.

The potential impact of PCN on potato yield from the in-depth survey of 86 fields in Nyandarua County showed that PCN were recovered from 72.1% of sampled fields and prevalent in all sub-counties. The highest incidence was observed in Kinangop (92.9%), followed by Kipipiri (80.0%), Ol’Joro Orok (78.6%), Ol’Kalou (52.9%) and Ndaragua (33.3%). Among these, infestation levels varied: between 1 < *X* < 25 cysts (24.4%), 26 < *X* < 110 cysts (22.1%), 111 < *X* < 230 cysts (14.0%), 231 < *X* < 495 cysts (5.8%), and 496 < *X* < 985 cysts (5.8%) respectively per 200 g soil. On average, higher infestation levels were found in Kinangop, with the largest number (66.7%) of highly infested fields or “hotspots” with cyst counts ≥231 cyst/200 g soil (Figures 3, 4).

The mean CV within Nyandarua County did not differ significantly among sub-counties, although mean CF levels were greater (*p* ≤ 0.05) in Kipipiri and Ol’Joro Orok; Kinangop and Kipipiri presented the highest SI levels (viable eggs/g soil) with 158.5 and 90.7 viable eggs/g soil, respectively, while the lowest SI levels were observed in Ol’Kalou (30.6 viable eggs/g soil). Yield losses attributed to PCN, considering the damage threshold of 2.75 t/ha per 20 viable eggs/g soil (Brown and Sykes, 1983), ranged from a minimum of 4.2 t/ha in Ol’Kalou to a maximum of 21.8 t/ha in Kinangop (Table 2) based on the SI data per sub-county.

**TABLE 2:** Potential yield losses calculated from Brown and Sykes (1983) formula using PCN cyst and egg data.

**Sub-county**	Mean number of cysts*	Mean cyst fertility (eggs/cyst)*	**Mean cyst viability (%)**	**Mean soil infectivity (viable egg/g soil)**	**Estimated mean yield loss (t/ha)**
Kinangop (*n* = 21)	215 ± 64.9	147.3 ± 7.6	77	158.5	21.8
Kipipiri (*n* = 15)	98 ± 34.9	183.4 ± 13.9	88	90.7	12.5
Ol’Joro Orok (*n* = 15)	48 ± 21.3	180.6 ± 13.1	82	42.3	5.8
Ol’Kalou (*n* = 20)	52 ± 31.6	127.6 ± 13.4	79	30.6	4.2
Ndaragua (*n* = 15)	46 ± 40.0	143.5 ± 22.9	67	37.3	5.1

*Values expressed as mean ± SE; Mean number of cysts recovered from 200 g soil sample.

### Smallholder Farmer Practices

The average household land size dedicated to potato production was 0.35 ha. Farmers acknowledged the use of up to 34 potato cultivars across Kenya (Table 3), among which cv. Shangi was the most preferred (65%, predominant in 13 counties), followed by cv. Arka (10%, predominant in Bungoma and Trans Nzoia Counties). Based on interviews, the overwhelming preference of cv. Shangi is attributed to its short dormancy (∼5–6 weeks) meaning that it does not require refrigeration/cool storage of tubers until the following planting season. It also has a fast cooking time, so it requires less fuel and labor for processing (i.e., boiling). This makes it particularly appealing to women, who are largely responsible for collection of firewood for cooking.

**TABLE 3:** Preferred potato cultivar grown in Kenyan counties and overall percentage uptake.

**County**	**Cultivar**	**Percentage**	**Overall%**
Bungoma	Arka	72.4	10
Trans Nzoia	Arka	70.0	
Taita Taveta	Asante	43.6	5
Bomet	Dutch Robjin	50.0	5
Laikipia	Humba Thuti	42.9	5
Embu	Kanyoni	30.0	5
Kirinyaga	Mukura Nooke	78.6	5
Baringo	Shangi	75.0	65
Elgeyo Marakwet	Shangi	92.6	
Kericho	Shangi	69.6	
Kiambu	Shangi	71.8	
Meru	Shangi	61.5	
Murang’a	Shangi	66.7	
Nakuru	Shangi	80.9	
Nandi	Shangi	77.8	
Narok	Shangi	89.6	
Nyandarua	Shangi	98.3	
Nyeri	Shangi	57.4	
Uasin Gishu	Shangi	92.9	
West Pokot	Shangi	100.0	

**FIGURE 5:**
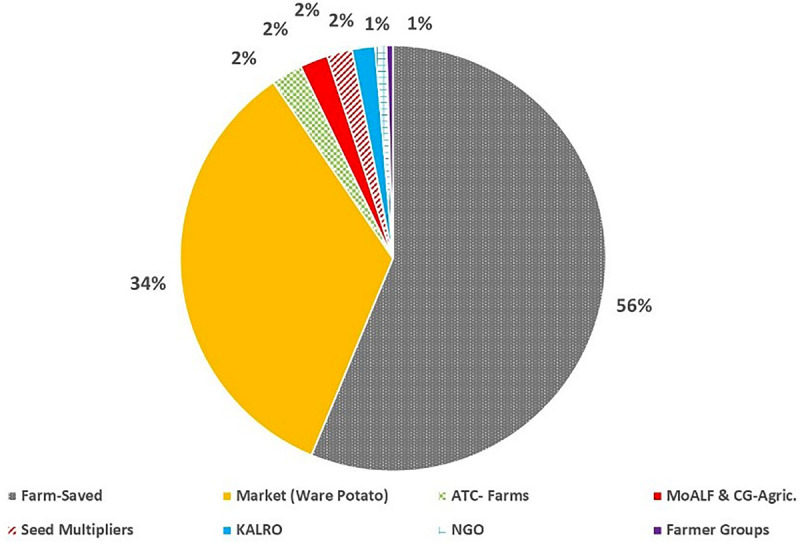
Source of planting material across the potato growing counties of Kenya.

**FIGURE 6:**
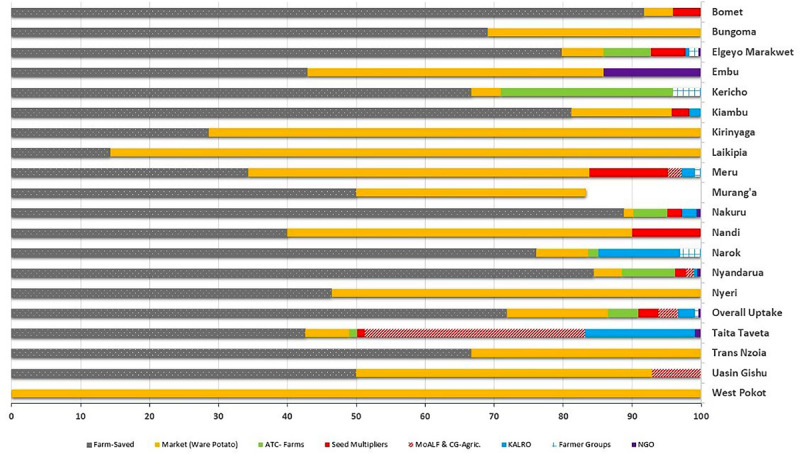
Diversity of seed sources within each county in all potato growing counties in Kenya.

Most farmers (56.0%; *n* = 752) identified farm-saved seed, which is recycled over several seasons, and ware-potatoes bought from local markets (34.0%; *n* = 458) as their principal source of planting material. Government and private institutional sources of seed, such as Agricultural Training College-Farms (ATCs) (2.3%), seed aid from the Ministry of Agriculture (2.1%), National Research Organizations (KALRO) (1.7%) or private certified seed multipliers (1.9%) accounted for just 8.9% of seed supply. Noteworthy, the diversity on the sources of potato seed used for planting also varied among counties, although the two informal sources for seed (“Farm-saved” and “Markets”) were predominant across counties (Figure 5). In counties such as Bungoma, Kirinyaga, Laikipia, Nyeri, and Trans Nzoia only “Farm-saved” and “Markets” seeds were acknowledged to be in use. The lowest diversity of potato seed source was observed in West Pokot, where “Markets” (ware potato) were the sole source of seed. The highest diversity of seed supply (*n* = 7) was recorded in Nyandarua and Taita Taveta. Only eight counties reported seed multipliers as an accessible source of planting material, and the highest percentage of farmers in Meru County (11.4%) accessed quality declared certified seeds (Figure 6). Respondents did not use certified seeds because of: (i) high cost of potato seeds; (ii) limited impact experienced in terms of increased yields; (iii) scarcity of certified seeds for the most-preferred cultivar (i.e., cv. Shangi) especially at the onset of planting season.

**FIGURE 7:**
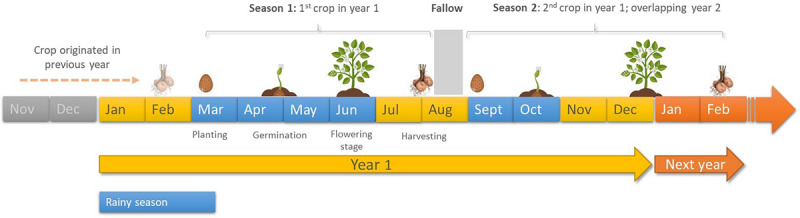
Scheme of potato cropping seasons in 1 year.

The majority of farmers (89%) indicated that they employed crop rotation to manage potato pests and diseases (Table 4). Thus, in six counties surveyed, 100% of farmers practiced rotation, while in the remaining 14 counties crop rotation was practiced by 60.0 to 93.1%. The majority of farmers practiced rotations over a period of >1 year (two cropping seasons) to 7 years (14 cropping seasons) after planting the first crop (72.4%), with one exception where farm-saved potato seeds had been cultivated successively in the same field for over 10 years (or at least 20 successive crops) (Figure 7). However, in all rotation schemes described by farmers, only 21.2% included a different crop immediately following the first potato crop.

**TABLE 4:** Adoption of crop rotation across the 20 surveyed counties in Kenya.

**County**	**No rotation**	**Rotation**	**Percentage**
Baringo	2	7	77.8
Bomet	3	23	88.5
Bungoma	2	27	93.1
Elgeyo Marakwet	78	143	64.7
Embu	0	9	100.0
Kericho	0	24	100.0
Kiambu	0	123	100.0
Kirinyaga	0	9	100.0
Laikipia	4	6	60.0
Meru	16	109	87.2
Murang’a	1	8	88.9
Nakuru	13	137	91.3
Nandi	1	9	90.0
Narok	7	62	89.9
Nyandarua	53	313	85.3
Nyeri	9	66	88.0
Taita Taveta	0	94	100.0
Trans Nzoia	1	8	88.9
Uasin Gishu	2	13	86.7
West Pokot	0	4	100.0

## Discussion

Recent studies reported for the first-time the occurrence of *G. rostochiensis* in Nyandarua County in Kenya (Mwangi et al., 2015). In the present study, we show that this PCN species is widespread and present in particularly high densities across the country, thus causing severe yield losses. The PCN species *G. pallida*, now confirmed in Kenya and sub-Saharan Africa (SSA) from the current study and separately documented as a new disease report (Mburu et al., 2018), is shown to be currently very restricted in its distribution. However, the country-wide distribution of *G. rostochiensis*, including counties bordering Uganda and Tanzania, where potato is also an important staple food source, creates great concern in respect to PCN distribution across the region. The recent report of *G. rostochiensis* in Rwanda (Niragire et al., 2019) demonstrates that the pest is already present and established elsewhere within the region.

During the current study, the inconsistency of positive amplifications to diagnose *G. rostochiensis* between the EPPO protocols (EPPO, 2013a, b) and the Bulman and Marshall (1997) protocol, indicates that false negatives could occur if only EPPO protocols had been used to diagnose Kenyan PCN populations. The Bulman and Marshall (1997) protocol has been commonly used in other parts of the world (Vossenberg et al., 2014), and specifically in detection of *G. rostochiensis* and *G. pallida* samples from Kenya (Mwangi et al., 2015; Mburu et al., 2018). Consequently, it is advised that at least two PCR-based protocols should be used to detect and diagnose PCN populations in the region and, importantly, that the EPPO protocol should not be entirely relied upon. However, the presence of other *Globodera* spp. other than PCN, in the country cannot be discounted either and should also be considered when receiving false positives.

In the East African region potato is a key commodity both for food security and as a cash crop for household income (Thiele et al., 2011). In Kenya, the crop represents a source of income for around 2.5 million people across the value chain (Abong and Kabira, 2013), with between 500,000 to 780,000 people directly involved in potato farming activities (Janssens et al., 2013). PCNs are highly destructive pests of potato, resulting in major losses to production and incurring significant investment toward their management (Niere and Karuri, 2018). They are indigenous to South America but have since become established in various potato growing regions around the world (Brodie et al., 1993; Hodda and Cook, 2009). In SSA, excluding South Africa, PCNs have been detected only in Kenya and Rwanda (Mwangi et al., 2015; Niragire et al., 2019), with a tenuous report from Sierra Leone (EPPO, 2009). The current study, therefore, determines that *G. rostochiensis* is now firmly established in the region and that *G. pallida* is present. The greater proportion of information on the importance of PCN has been established from studies in temperate climates. Kenya has a sub-tropical climate with an absence of prolonged frosting periods and relatively mild minimum temperatures across the year. This provides favorable conditions for 2–3 potato cropping seasons per year, given enough rainfall or irrigation (Gildemacher et al., 2009). The current study provides the first indication of PCN damage potential under such agro-ecological conditions, which provides compelling justification that urgent and serious action is needed. Our current study demonstrates that PCN is seriously threatening potato production in Kenya, where extremely high infestation levels are present. Current data indicate that on average, potato yields are equivalent to 9.9 t/ha, or 24.75% of the potential yield (40 t/ha). This equates to approximately USD $127 million annual potato losses in Kenya, based on two cropping seasons per year of susceptible cultivars, such as cv. Shangi. Even though the damage threshold used to calculate production losses in the current study were based on data from Europe (Brown and Sykes, 1983; EPPO, 2013a), this extrapolation is considered a realistic indication of the magnitude of PCN damage to potato production in Kenya. The pervasive presence and high densities of PCN across the country undoubtedly helps to explain some of the current potato yield gaps, and why these have been increasing over recent years. Given the persistent shortage of certified seed potatoes and the inherent nature of using farm-saved seed, as highlighted from the current study, potato production gaps are likely to further widen due to low quality of planting material, other pest and diseases, or inefficient post-harvest handling and storage conditions (Muthoni et al., 2013). During the current study, PCN was established in ∼82% of the seed production farms in Kenya. These findings challenge the status of the formal seed system and highlights the urgent need for comprehensive phytosanitary control measures to be implemented in commercial potato seed operations (Cortada, 2018).

Plant parasitic nematodes are, over time, repeatedly overlooked as damaging soil-borne pests in Africa (Coyne et al., 2018). Their subterranean habit and often indistinct damage symptoms contribute to this neglect, and lack of awareness. Given that PCN are readily disseminated through the use of infected potato seed, the routine use of farm-saved tubers for seed in Kenya (Abong and Kabira, 2013) and the region (Gildemacher et al., 2009) undoubtedly encourages and perpetuates the PCN issue. Such inherent lack of awareness across the agricultural spectrum has inevitably played its part in facilitating its spread. How long PCN has been present and how it was introduced remains open to question. Potato was first introduced to Africa in the 17th century by Christian missionaries (VIB, 2019), while evidence of their importation into Kenya dates back over 100 years for agricultural diversification, seed aid (The Kenya Times, 1985) and for research purposes (McArthur, 1989). If we consider the sub-tropical climate, lack of frosting periods, year-round production with multiple cropping seasons per annum of susceptible cultivars, as well as the habitual use of farm-saved seed, the introduction of PCN may not have been as long ago as the high infestation levels would suggest (Nyongesa, 2015). The high mean cyst density levels of 98 cysts/200 cc soil (0.49 cysts/cc soil) and up to 985 cysts/200 cc in Kenya, which equate to 980 million cysts/ha, is way above the official detection threshold of 3.8 million cysts/ha in Europe (Spears, 1968; Davie et al., 2017), with mature and female cysts visible to the naked eye attached to roots. The densities encountered are consequently remarkably high and way above European norms. The agroecological factors in Kenya have therefore quite likely supported the “rapid” dispersal of PCN and development of such high densities. It is quite possible that the physiological nature and hatching behavior of these Kenya populations differ from their temperate climate counterparts. For example, the optimum temperature for hatch of the cereal cyst nematode, *Heterodera avenae*, recovered from Egypt was on average 5°C higher than for *H. avenae* populations recovered from Germany (Baklawa et al., 2017). This information is important to determine, as successful management options can be highly dependent on such characteristics.

In line with previous findings (Kaguongo et al., 2014; Sinelle, 2018), farmers interviewed in our study identified cv. Shangi as the most important and preferred potato cultivar. This farmer-selected cultivar was rapidly and informally introduced and distributed in the early 2000s (ISSD Africa, 2016) before its official release (Sinelle, 2018). Its short dormancy and fast sprouting nature makes it appealing and a ready source of recycled seeds that do not require cold storage between planting seasons. It was quickly adopted by Kenyan farmers, thus replacing other potato cultivars due to increasing market demand, early maturity and high yielding, despite its susceptibility to pests and diseases, particularly late blight. To our knowledge, our study is the first report confirming the susceptibility of cv. Shangi to *G. rostochiensis* infection. Following the preliminary results of our study in 2017 (FAO, 2017a; icipe, 2017), PCN testing was made a mandatory procedure for seed certification schemes, with resistance considered necessary for potato cultivars (NPCK, 2019). Consequently, the identification and introduction of cultivars with similar attributes to cv. Shangi, but with resistance against PCN could prove highly beneficial to addressing the PCN problem in Kenya, and the region. In addition, although farmers considered crop rotations as a suitable cultural practice to control pests and diseases, they were unaware of the importance of establishing suitable (non-PCN hosts) and sufficiently long rotation schemes to manage PCN. Smallholder farmers, who represent 98% of potato growers in Kenya, primarily grow potato as a cash crop, and for home consumption. They produce 83% of the national production, with an average farm size dedicated to this crop of less than 0.4 ha/year (Janssens et al., 2013). A lack of suitable land coupled with the prevailing status of potato as an income-generating cash crop deters farmers from practicing fallow or cultivating less lucrative non-host crops. A situation arises therefore with the continued cultivation of potato in infested farms until potato production becomes unprofitable. Awareness campaigns are therefore urgently required to enable farmers to understand the importance of managing this aggressive pest. As per international regulations (IPPC, 2008), countries affected by PCN are required to adopt strict phytosanitary regulations for seed potato production to prevent its further spread within and outside their borders. The results of the current epidemiological study on PCN in Kenya provide a basis upon which to establish suitable management approaches and policies against this destructive nematode pest.

## Conclusion

Our results show that PCN is widely spread across the main potato growing counties of Kenya. *Globodera rostochiensis* is widely distributed, while *G. pallida* is currently highly restricted but present. More robust sampling techniques will be required to ascertain that fields are free of PCN infestation, rather than having low, below-threshold detection levels. Development of a suitable tool to validate the estimation of yield losses under Kenyan conditions is urgently required. There is need to create awareness with farmers on the importance of using certified seed, adoption of PCN resistant cultivars and practicing crop rotations. Due to inconsistencies in results from established molecular protocols, an in-depth molecular analysis of the PCN from Kenya would be required to fully characterize and understand the genetic complexity of cyst nematode populations present, including PCN pathotypes. Given the sub-tropical nature prevailing in potato production areas, it will also be important to establish if this has influenced the phenotypic and behavioral nature of PCN in Kenya, which will have significant implications to the development of management options.

## Data Availability Statement

Reference sequences used in this study for phylogenetic analysis are freely available at the NCBI database (https://www.ncbi.nlm.nih.gov/) under the following accession numbers: MG309873.1, MG309920.1, MN382342.1 – MN382349.1, MN378550.1, MN378566.1, and MN378644.1.

## Author Contributions

HM, SH, LC, WR, MN, and ZK designed the study. LC and HM analyzed the data. DC, SH, MN, LC, JB, and HM wrote the manuscript. All authors improved and approved the final manuscript.

## Conflict of Interest

The authors declare that the research was conducted in the absence of any commercial or financial relationships that could be construed as a potential conflict of interest.

## Footnotes

1 http://www.epicollect.net/

## References

[B1] Abong G. O. Kabira J. N. (2013). “The current status of the potato value chain in Kenya,” in *Trends and Opportunities in the Production, Processing and Consumption of Staple Foods Crops in Kenya*, eds Onyango C. Unbehend G. Lindhauer M. (Dresden: TUD Press), 56–59.

[B2] Baklawa M. Niere B. Massoud S. (2017). Influence of temperature and storage conditions on the hatching behavior of cereal cyst nematodes (*Heterodera avenae* Wollenweber) from Egypt. *J. Plant Dis.Prot.* 124 213–225.

[B3] Brodie B. B. Evans K. Franco J. (1993). “Nematode parasites of potatoes,” in *Plant Parasitic Nematodes in Temperate Agriculture*, eds Evans K. Trudgill D. L. Webster J. M. (Wallingford: Cab International), 87–132.

[B4] Brown E. B. Sykes G. B. (1983). Assessment of the losses caused to potatoes by the potato cyst nematodes, *Globodera rostochiensis* and *G. pallida*. *Ann. Appl. Biol.* 103 271–276. 19270925

[B5] Bulman S. R. Marshall J. W. (1997). Differentiation of Australasian potato cyst nematode (PCN) populations using the polymerase chain reaction (PCR). *N. Z. J. Crop Hortic. Sci.* 25 123–129.

[B6] CIP (2019). *Farming Success with Potatoes in Kenya.* Available online at: https://cipotato.org/media/farming-success-potatoes-kenya/2019 (accessed November 5, 2019).

[B7] Cortada L. (2018). *Outcome Case Overview. Roots, Tubers and Bananas (RTB) Report.* 1–4.

[B8] Coyne D. L. Nicol J. M. Claudius-Cole B. (2018). *Practical Plant Nematology: A Field and Laboratory Guide.* Cotonou: SP-IPM Secretariat, International institute of Tropical Agriculture (IITA), 82.

[B9] Davie K. Pickup J. Cole Y. (2017). *Potato Cyst Nematode Management in Scotland.* 1–31.

[B10] Devaux A. Kromann P. Ortiz O. (2014). Potatoes for sustainable global food security. *Potato Res.* 57 185–199.

[B11] Devine K. J. Dunne C. O’Gara F. Jones P. W. (1999). The influence of in-egg mortality and spontaneous hatching on the decline of *Globodera rostochiensis* during crop rotation in the absence of the host potato crop in the field. *Nematology* 1 637–645.

[B12] EPPO (2009). PM 7/40(2) *Globodera rostochiensis* and *Globodera pallida*. *Bull. OEPP* 39 354–368.

[B13] EPPO (2013a). PM 7/40(3) Erratum *Globodera rostochiensis* and *Globodera pallida*. *Bull. OEPP* 43 564–564.

[B14] EPPO (2013b). PM 7/40(3) *Globodera rostochiensis* and *Globodera pallida*. *Bull. OEPP* 43 119–138.

[B15] EPPO (2017). PM 7/40(4) *Globodera rostochiensis* and *Globodera pallida*. *Bull. OEPP* 47 174–197.

[B16] Faggian R. Powell A. Slater A. T. (2012). Screening for resistance to potato cyst nematode in Australian potato cultivars and alternative solanaceous hosts. *Australas. Plant Pathol.* 41 453–461.

[B17] FAO (2017a). *Combating Potato Cyst Nematode (PCN) in Kenya.* Rome: FAO publications, 1–2.

[B18] FAO (2017b). *The State of Food and Agriculture. Leveraging Food for Inclusive Rural Transformation.* Rome: FAO publications.

[B19] FAOSTAT (2018). *Food and Agricultural Organization of the United Nations. FAO Statistical Database.* Available online at: http://www.fao.org/faostat/en/#data/QC (accessed September 11, 2018).

[B20] Folkertsma R. T. Helder J. Gommers F. J. Bakker J. (1997). Storage of potato cyst nematode at −80°C. *Fund. App. Nematol.* 20 299–302.

[B21] Gildemacher P. R. Demo P. Barker I. Kaguongo W. Woldegiorgis G. Wagoire W. W. (2009). A description of seed potato systems in Kenya, Uganda and Ethiopia. *Am. J. Potato Res.* 86 373–382.

[B22] Goeminne M. Demeulemeester K. Lanterbecq D. Proft M. D. Viaene N. (2015). Detection of field infestations of potato cyst nematodes (PCN) by sampling soil from harvested potatoes. *Asp. Appl. Biol.* 130 105–110. 10.1007/978-1-4939-2620-6_11 25981252

[B23] Goeminne M. Demeulemeester K. Viaene N. (2011). A method for estimating the contribution of seed potatoes, machinery and soil tare in field infestations with potato cyst nematodes on a national scale. *Commun. Agric. Appl. Biol. Sci.* 76 311–318. 22696943

[B24] Gouy M. Guindon S. Gascuel O. (2010). SeaView version 4: a multiplatform graphical user interface for sequence alignment and phylogenetic tree building. *Mol. Biol. Evol.* 27 221–224. 10.1093/molbev/msp259 19854763

[B25] Hall T. A. (1999). BioEdit: a user-friendly biological sequence alignment editor and analysis program for Windows 95/98/NT. *Nucl. Acids. Symp. Ser.* 41 95–98.

[B26] Harahagazwe D. Condori B. Barreda C. Bararyenya A. Byarugaba A. A. Kude D. A. (2018). How big is the potato (*Solanum tuberosum* L.) yield gap in Sub-Saharan Africa and why? A participatory approach. *Open Agric.* 3 180–189.

[B27] Haverkort A. J. Struik P. C. (2015). Yield levels of potato crops: recent achievements and future prospects. *Field Crops Res.* 182 76–85.

[B28] Hodda M. Cook D. C. (2009). Economic impact from unrestricted spread of potato cyst nematodes in Australia. *Phytopathology* 99 1387–1393. 10.1094/PHYTO-99-12-1387 19900005

[B29] icipe (2017). *Training of 20 Government Technicians and Determination of PCN Status in 1,200 Soil Samples. (Icipe Final Narrative. Report., Icipe, Nairobi, Kenya).* Available online at: http://www.icipe.org/research/plant-health/research-nematodes/projects/fao-emergency-assistance-control-potato-cyst

[B30] IPPC (2008). *Extension of Known Potato Cyst Nematode (PCN) in Victoria, Australia.* (IPPC Official Pest Report, Rome, Italy: FAO). No. AU-16/1. Available online at: https://www.ippc.int/IPP/En/default (accessed August 15, 2019).

[B31] ISSD Africa (2016). *The Role of Variety User Agreements in Access of Public Potato (Solanum tuberosum) Varieties in Kenya.* Available online at: http://www.issdseed.org/sites/default/files/alp_4_final_report_potato_user_agreements_final.pdf (accessed June 13, 2019).

[B32] Jaetzold R. Schmidt H. (1983). *Farm Management Handbook of Kenya RRC-Embu. Vol. II/B.* Nairobi: Ministry of Agriculture.

[B33] Janssens S. R. M. Wiersema S. G. Goos H. Wiersma W. (2013). *The Value Chain for Seed and Ware Potatoes in Kenya Opportunities for Development*. LEI Memorandum 13-080. LEI Wageningen UR, Den Haag, NL). Den Haag: LEI Wageningen UR, 57.

[B34] Joyce S. A. Burnell A. M. Powers T. O. (1994). Characterization of *Heterorhabditis* isolates by PCR amplification of segments of mtDNA and rDNA genes. *J. Nematol.* 26 260–270. 19279891 PMC2619499

[B35] Kaguongo W. Maingi G. Giencke S. (2014). *Post-harvest Losses in Potato Value Chains in Kenya: Analysis and Recommendations for Reduction Strategies*, eds Lohr K. Pickardt T. Ostermann H. (Bonn: Deutsche Gesellschaft für Internationale Zusammenarbeit (GIZ) GmbH), 80.

[B36] Kiptoo K. W. Xia X. Kipkemboi K. K. (2016). Technical efficiency of smallholder potato farmers in Kenya: an analysis of major influencing factors in Koibatek, Baringo County. *Afri. J. Agric.Environ.* 2 8–15.

[B37] Mburu H. Cortada L. Mwangi G. Gitau K. Kiriga A. Kinyua Z. (2018). First report of potato cyst nematode *Globodera pallida* infecting potato (*Solanum tuberosum*) in Kenya. *Plant Dis.* 102 1671–1671.

[B38] McArthur C. L. (1989). *Evaluation, Choice and use of Potato Varieties in Kenya. Social Science Department, Working Paper 1989-1.* Lima: CIP, 44.

[B39] Mimee B. Dauphinais N. Bélair G. (2019). “Piler Dirt” survey for the sampling and detection of potato cyst nematodes. *Plant Dis.* 103 2065–2069. 10.1094/PDIS-12-18-2188-RE 31169084

[B40] Muthoni J. Shimelis H. Melis R. (2013). Potato production in Kenya: farming systems and production constraints. *J. Agric. Sci.* 5 182–197.

[B41] Mwangi J. Waceke J. Kariuki G. Grundler F. (2015). First Report of *Globodera rostochiensis* infesting potatoes in Kenya. *New Dis. Rep.* 31:18.

[B42] Niere B. Karuri H. (2018). “Nematode parasites of potato and sweet potato,” in *Plant Parasitic Nematodes in Subtropical and Tropical Agriculture*, eds Sikora R. Coyne D. Hallmann J. Timper P. (Wallingford: CAB International), 222–251.

[B43] Niragire I. Couvreur M. Karssen G. Uwumukiza B. Bert W. (2019). First report of potato cyst nematode (*Globodera rostochiensis*) infecting potato (*Solanum tuberosum* L.) in Rwanda. *Plant Dis.* 104:293.

[B44] NPCK (2017). *National Potato Council of Kenya. Potato Variety Catalogue.* Available online at: https://npck.org/Catalogues/NPCKCATALOGUE2017bookletK2.pdf (accessed August 20, 2019).

[B45] NPCK (2019). *National Potato Council of Kenya. Potato Variety Catalogue.* Available online at: https://npck.org/Catalogues/NPCKOnlineDocument.pdf (accessed August 20, 2019).

[B46] Nyongesa M. (2015). What you need to know about potato cyst nematode (PCN), a new threat to potato farming in Kenya. *HortiNews* 38:44.

[B47] Official Journal of European Union (2007). *Council Directive 2007/33/EC of 11 June 2007. L 156, 12–22.* Available online at: https://eur-lex.europa.eu/eli/dir/2007/33/oj (accessed June 11, 2007).

[B48] Ogiga I. R. Estey R. H. (1974). The use of meldola blue and nile blue A, for distinguishing dead from living nematodes. *Nematologica* 20 271–276.

[B49] Okello J. J. Zhou Y. Kwikiriza N. Ogutu S. Barker I. Shulte-Geldermann E. (2017). Productivity and food security effects of using of certified seed potato: the case of Kenya’s potato farmers. *Agric. Food Secur.* 6 25–34.

[B50] Park J. Yang H. De Jong W. S. Wang X. (2018). An evaluation of two *H1*-linked markers and their suitability for selecting *Globodera rostochiensis* resistant potatoes in the New York breeding program. *Am. J. Potato Res.* 95 170–177.

[B51] Perry R. N. (2002). “Hatching,” in *Biology of Nematodes*, ed. Lee D. L. (London: Taylor and Francis), 147–169.

[B52] Reader J. (2009). *The Untold History of the Potato.* London: Vintage, 315.

[B53] Scott G. J. Labarta R. Suarez V. (2013). Booms, busts, and emerging markets for potatoes and potato products in East and Central Africa 1961-2010. *Potato Res.* 56 205–236.

[B54] Scott G. J. Suarez V. (2012). From Mao to McDonald’s: emerging markets for potatoes and potato products in China 1961–2007. *Am. J. Potato Res.* 89 216–231.

[B55] Sinelle S. (2018). *Potato Variety Adoption and Dis-adoption in Kenya.* Nairobi: CIP and Syngenta Foundation.

[B56] Spears J. F. (1968). The golden nematode handbook: survey, laboratory, control, and quarantine procedures. *US Agric. Res. Serv.* 353 1–86.

[B57] The Kenya Times (1985). *Farmers Cry Out: Where is the Rain? Press Article(Monday, March 18, 1985.* Nairobi: The Kenya Times.

[B58] Thiele G. Labarta R. Schulte−Geldermann E. Harrison G. (2011). *Roadmap for Investment in the Seed Potato Value Chain in Eastern Africa.* Lima: International Potato Center, 27.

[B59] VIB (2019). *Potato in Africa. Fact Sheets.* Available online at: http://www.vib.be/VIBDocumentLibrary/VIB_Facts%20Series_Potato%20in%20Africa%20LR.pdf (accessed June 6, 2019).

[B60] Vossenberg B. Voogd J. Westenberg M. Karssen G. (2014). Comparison of three conventional PCR test (Bulman and Marshall) versions for the molecular identification of *Globodera pallida* and *G. rostochiensis* cysts and juveniles. *EPPO Bull.* 44 27–33.

