# Supplementary material for: Potato Cyst Nematodes: A New Threat to Potato Production in East Africa
Source: Front Plant Sci. 2020 May 25;11:670. doi: 10.3389/fpls.2020.00670 (PMC7261874; doi:10.3389/fpls.2020.00670)
Supplement: Supplementary file 1 [file Table_1.DOCX]

Supplementary Material

**Potato Cyst Nematode Sampling Form**

Team members should introduce themselves and state clearly the objective(s) of their visit to a farm, mode and purpose of sampling and request to be allowed to do so.

| **Date** | | | **Interview start time** | | | | | | | | | | **Survey Form No.** | | | |
| --- | --- | --- | --- | --- | --- | --- | --- | --- | --- | --- | --- | --- | --- | --- | --- | --- |
| **GPS Coordinates** | | | | | | | |  | **Administrative location** | | | | | | | |
| Latitude | | | | | | | |  | County | | | | | | | |
| Longitude | | | | | | | |  | Sub-County or  Constituency | | | | | | | |
| Altitude (m) | | | | | | | |  | Location or  Ward | | | | | | | |
| Nearest town or shopping centre | | | | | | | |  | Sub-Location or  Village | | | | | | | |
| Interviewee’s Name | | | | | | | |  | | | | | | | | |
| Relationship with farm | | | | | | | |  | | | | | | | | |
| Cell Phone number (or other contact) | | | | | | | |  | | | | | | | | |
| Which crops Solanaceous crops are normally grown on this farm? | | | | | | | | | | | | | | | | |
| Tick in appropriate space | | Potato | | | | | Tomato | | | Capsicum | | | Others (write the name of the specifics crop) | | | |
| Yes | |  | | | | |  | | |  | | |  | | | |
| No | |  | | | | |  | | |  | | |  | | | |
| If potatoes are normally grown on this farm, state the following: | | | | | | | | | | | | | | | | |
| Main varieties grown (list names) | | | | Area normally planted (acres) | | | | | | Source(s) of planting material | | | | | Other comments | |
| 1. | | | |  | | | | | |  | | | | |  | |
| 2. | | | |  | | | | | |  | | | | |  | |
| 3. | | | |  | | | | | |  | | | | |  | |
| Does the interviewee do crop rotation in potato fields | | | | | | | | | | | | | Yes (tick) ----- | | No (tick) ----- | |
| If Yes, give length of rotation period  (No. of crop seasons) | | | | | | | | | |  | State crops used in rotation with potato | | | | |  |
| Has the interviewee ever seen symptoms/signs of potato cyst nematodes? (show photographs) | | | | | | | | | | | | | | | | |
|  | Yes (tick) | | | | No (tick) | If yes, when were the symptoms or signs seen for the first time? | | | | | | What effects are attributable to the symptoms or signs | | | | |
| On potato |  | | | |  |  | | | | | |  | | | | |
| On capsicum |  | | | |  |  | | | | | |  | | | | |
| On tomato |  | | | |  |  | | | | | |  | | | | |
| Others (specify) |  | | | |  |  | | | | | |  | | | | |
|  | | | | No. | | | Description of sampled land | | | | | | | Other notes (e.g. photos take, drawing a sketch map of the sampled land overleaf, etc) | | |
| Soil samples taken for PCN | | | |  | | |  | | | | | | |  | | |
| Plant samples taken for PCN | | | |  | | |  | | | | | | |  | | |
| **Interview end time** | | | |  | | | **Name of interviewer** | | | | | |  | | | |
